# Explanatory Cognitive Diagnosis Models Incorporating Item Features

**DOI:** 10.3390/jintelligence12030032

**Published:** 2024-03-11

**Authors:** Manqian Liao, Hong Jiao, Qiwei He

**Affiliations:** 1 Duolingo, Inc., 5900 Penn Ave, Pittsburgh, PA 15206, USA; 2 Department of Human Development and Quantitative Methodology, Maryland Assessment Research Center (MARC), University of Maryland, College Park, MD 20742, USA; hjiao@umd.edu; 3 Data Science and Analytics Program, Georgetown University, Washington, DC 20057, USA

**Keywords:** cognitive diagnosis model, explanatory model, linear logistic test model, item features, text mining

## Abstract

Item quality is crucial to psychometric analyses for cognitive diagnosis. In cognitive diagnosis models (CDMs), item quality is often quantified in terms of item parameters (e.g., guessing and slipping parameters). Calibrating the item parameters with only item response data, as a common practice, could result in challenges in identifying the cause of low-quality items (e.g., the correct answer is easy to be guessed) or devising an effective plan to improve the item quality. To resolve these challenges, we propose the item explanatory CDMs where the CDM item parameters are explained with item features such that item features can serve as an additional source of information for item parameters. The utility of the proposed models is demonstrated with the Trends in International Mathematics and Science Study (TIMSS)-released items and response data: around 20 item linguistic features were extracted from the item stem with natural language processing techniques, and the item feature engineering process is elaborated in the paper. The proposed models are used to examine the relationships between the guessing/slipping item parameters of the higher-order DINA model and eight of the item features. The findings from a follow-up simulation study are presented, which corroborate the validity of the inferences drawn from the empirical data analysis. Finally, future research directions are discussed.

## 1. Introduction

It is a common practice that the item parameters of the cognitive diagnosis models (CDMs) are calibrated only using the item response data. Such practice could result in challenges in item development. For example, when the item parameter estimate(s) suggest that an item is of low quality (e.g., the correct answer is easy to be guessed), it is hard to identify the cause of such low quality or to devise an effective item revision plan to improve the item quality.

Similar issues were encountered in the Item Response Theory (IRT) framework as well where the item parameters were traditionally estimated solely by the response data. Fortunately, the availability of the item features has provided viable solutions to both issues in the IRT framework. In particular, the item linguistic features were found to be associated with the item difficulty (e.g., Drum et al. 1981; Embretson and Wetzel 1987; Jerman and Mirman 1973; Lepik 1990) and served as an additional piece of information to explain and inform the IRT model parameters (e.g., Embretson and Wetzel 1987; Paap et al. 2015). In the item explanatory IRT models proposed by De Boeck and Wilson (2004), the observed item features were included in the traditional IRT models to explain the item parameters that had traditionally been descriptive. A well-known instance of the item explanatory IRT model is the linear logistic test model (LLTM; Fischer 1973) where the item difficulty parameter of the Rasch model is explained by some item features.

The additional information provided by item features could be even more valuable in the CDM framework than in the IRT framework. The item parameters of the CDMs (e.g., guessing and slipping probabilities) could be less straightforward and harder to manipulate in the item writing process than those in the IRT models (e.g., item difficulty), which calls for more pressing needs of explaining these CDM item parameters with manifest item features. However, to date, no studies have been performed to link the item features to the item parameters of the CDMs, despite the fact that some emerging explanatory CDMs have linked some person covariates to the CDM person or the structural parameters (Ayers et al. 2013; Park et al. 2018; Park and Lee 2014, 2019). Historically, a possible obstacle to incorporating the item features in the measurement models could be the fact that the item feature extraction tasks used to be costly in time and human resources. For example, the item features may need to be analyzed and be coded manually by multiple groups of readers (e.g., Drum et al. 1981).

To fill the gap in the research on the CDMs with item features, we propose the item explanatory CDMs that explain the CDM item parameters with item features. We also took advantage of natural language processing (NLP), which makes it feasible to extract the item features efficiently. The most direct implication of the proposed models is that they reveal the relationships between the descriptive CDM item parameters (particularly the guessing and slipping parameters in this study) and the manifest item linguistic features. Understanding such explanatory relationships could further shed light on the item revision to improve item quality. The rest of the paper is structured as follows: After establishing the theoretical framework and detailing the specifications of the proposed models, we demonstrate their application using the Trends in International Mathematics and Science Study (TIMSS) data. We particularly focus on explaining item parameters with item features. To assist future researchers, the process of item feature engineering is detailed. The robustness of our empirical data analysis is supported by a simulation study, which evaluates model parameter recovery under various feature configurations.

## 2. Theoretical Framework

### 2.1. Explanatory CDM

CDMs are a type of models that provide inferences on people’s strengths and weaknesses on a series of attributes. Rupp et al. (2010) have mentioned the feasibility of including covariates into the structural portion of the CDMs. In recent decades, some studies have been conducted to incorporate person covariates to explain the model parameters in the CDMs (Ayers et al. 2013; Park et al. 2018; Park and Lee 2014, 2019; Templin 2004). In particular, Ayers et al. (2013) and Park and Lee (2014) have linked the observed person covariates to either single attribute or response probabilities in the deterministic inputs, noisy-and-gate (DINA; Haertel 1989; Macready and Dayton 1977) model using a logistic function. More recently, Park et al. (2018) proposed an explanatory CDM (ECDM) framework that incorporated latent covariates, in addition to the observed ones, to explain the attribute profiles or item responses in the re-parameterized DINA model. However, while Park et al. (2018) extensively explored the incorporation of observed or latent person predictors (e.g., confidence), they did not delve into the use of item predictors (e.g., item characteristics) or the explanation of the item parameters.

### 2.2. Linking Item Features to Item Psychometric Properties

Manifest item features, especially linguistic features, have been found to be related to the item *p*-value—a difficulty index in the Classical Test Theory (CTT)—for a variety of educational problems, such as reading comprehension (Drum et al. 1981), arithmetic word problems (Jerman and Mirman 1973) and algebraic word problems (Lepik 1990). Some more recent studies based on the IRT framework have also identified some item linguistic features to be significant predictors of item or assessment properties such as item difficulty (Embretson and Wetzel 1987) and testlet dependency (Paap et al. 2015). Thus, it is worth exploring whether the manifest item features, especially linguistic features, are associated with the item psychometric properties in the CDM framework.

## 3. The Proposed Model

### 3.1. Model Specification

The proposed models differ from existing explanatory CDMs (Ayers et al. 2013; Park et al. 2018; Park and Lee 2014, 2019; Templin 2004) by focusing on explaining CDM item parameters using item features. This distinction arises from two main perspectives: utility and model formulation. Unlike the existing models, which primarily enhance the estimation and explanation of person attribute profiles or item responses, the proposed models utilize item predictors to explain and potentially enhance the estimation of item parameters. This implies that the audience or users differ between the existing explanatory CDMs and the proposed models. Existing models could be primarily oriented towards educators and policymakers. They provide insights into person attributes, aiding in the allocation of educational resources or tailoring instructional strategies to enhance student skills. On the other hand, the proposed models are designed with test developers and item writers in mind. These users benefit from understanding how specific item features influence item parameters, thus aiding in the creation and refinement of assessment items for improved quality and effectiveness. From the model formulation standpoint, while existing models link covariates (mostly person-related) with person parameters or directly to item responses, the proposed models distinctly and separately associate item covariates with item parameters, introducing greater flexibility in the use of the item covariates

In line with De Boeck and Wilson (2004)’s terminology, we view the existing explanatory CDMs, including the ECDMs proposed by Park et al. (2018), as more aligned with explanatory models on the person side. In contrast, the proposed models are conceptualized as explanatory models on the item side. More specifically, we position the proposed models as a CDM variation of the LLTM. Furthermore, we explored the integration of a random effect in item parameters, analogous to the random effect extension in LLTM (Janssen et al. 2004).

Since this is the first attempt to explain the item parameters of the CDMs with item covariates, the DINA model (Junker and Sijtsma 2001; Macready and Dayton 1977) is chosen as the foundation of the proposed models due to its popularity and simplicity, thereby enabling this paper to focus on the innovative explanatory part of the model. However, it would be straightforward to extend the proposed models to more generalized CDMs, such as the LCDM (Henson et al. 2009) and the G-DINA model (de la Torre 2011), by linking the item features to the interested item parameters in these models (e.g., item intercept and attribute main effects). The DINA model specifies that the probability of a correct item response as

$$P\left(Y_{ij}=1|\eta_{ij},g_{i},s_{i}\right)={\left(1-s_{i}\right)}^{\eta_{ij}}{\left(g_{i}\right)}^{1-\eta_{ij}},$$

where $\eta_{ij}=\prod_{k=1}^{K}\alpha_{jk}^{q_{ik}}$, indicating whether person *j* masters all attributes required to solve item *i*; $\alpha_{jk}$ indicates whether person *j* masters attribute *k*; and $q_{ik}$ indicates whether item *i* requires attribute *k* in the Q-matrix. $g_{i}$ and $s_{i}$ are guessing and slipping parameters, respectively, which are also treated as item parameters. Items with higher guessing and slipping parameters are usually considered to be of lower quality (Ma et al. 2016) and, thus, could reduce the classification accuracy (de la Torre et al. 2010; Kaplan 2016; Ma et al. 2016; Sorrel et al. 2017). The joint likelihood of the DINA model is given as

$$L\left(s,g;\eta \right)=\prod_{j=1}^{J}\prod_{i=1}^{I}{\left[s_{i}^{1-y_{ij}}{\left(1-s_{i}\right)}^{y_{ij}}\right]}^{\eta_{ij}}{\left[g_{i}^{y_{ij}}{\left(1-g_{i}\right)}^{1-y_{ij}}\right]}^{\eta_{ij}},$$

where $y_{ij}$ indicates whether person j responds to item i correctly.

Analogous to the LLTM, the proposed item explanatory DINA models extend the DINA model by decomposing its guessing or slipping parameters into a linear combination of item features through a logit link function, i.e., $\mathrm{logit}\left(g_{i}\right)=\gamma_{0}+\sum_{m=1}^{M}\gamma_{m}Z_{im}$ or $\mathrm{logit}\left(s_{i}\right)=\varphi_{0}+\sum_{m=1}^{M}\varphi_{m}Z_{im}$, where $Z_{i}$ is a vector that contains *M* item features of item *i*; $\gamma_{0}$ and $\varphi_{0}$ are intercepts (i.e., the logit scale of the guessing or slipping probabilities when all the item features take the value of 0); and $\gamma_{m}$ and $\varphi_{m}$ are coefficients of the *m*th item feature. The above specifications of the explanatory component have assumed that the item features can perfectly predict the item parameters, which could be an overly strong assumption. Alternatively, a residual term can be included in the model to absorb the unexplained variance in the item parameters, i.e., $\log it(g_{i})=\gamma_{0}+\sum_{m=1}^{M}\gamma_{m}Z_{im}+\varepsilon_{(g)i}$, or $\log it(s_{i})=\varphi_{0}+\sum_{m=1}^{M}\varphi_{m}Z_{im}+\varepsilon_{(s)i}$.

We choose to use the Bayesian Markov chain Monte Carlo (MCMC) method to estimate the parameters of the proposed models, as it has proved to be useful and, in theory, superior to the frequentist methods in estimating the LLTM+e model (i.e., the LLTM with a residual term) which treated both the person and item parameters as random effects (De Boeck 2008; Janssen et al. 2004). Accordingly, we assume a general unidimensional factor $\theta_{j}$ underlying the attributes, i.e., $P(\alpha_{jk}^{\;}=1|\theta_{j})=\frac{\exp(\xi_{k}\theta_{j}\;+\;\beta_{k})}{1\;+\;\exp(\xi_{k}\theta_{j}\;+\;\beta_{k})}$, as such higher-order structure could improve the estimation efficiency of the Bayesian MCMC method in CDMs (de la Torre and Douglas 2004).

To accurately describe the nature of the proposed models, the proposed models are referred to as the item explanatory higher-order DINA (IE-HO-DINA) models in the subsequent sections. In summary, four IE-HO-DINA models are proposed, and they vary on (1) the item parameter (i.e., whether the guessing or the slipping parameter) that is linked to the item features and (2) whether an item residual term is included. The four models are labelled as IE-HO-DINA-g, IE-HO-DINA-s, IE-HO-DINA-g-R and IE-HO-DINA-s-R, and their detailed specifications can be found in Table 1. Note that since the originally item-specific guessing and slipping parameters are expressed in more canonical forms in the IE-HO-DINA models, i.e., as combinations of item features, the IE-HO-DINA models are expected to be more reduced and have fewer item parameters than the HO-DINA models.

### 3.2. Model Constraints and Identification

Two major constraints are needed to ensure the identification of the IE-HO-DINA models. First, the mean and variance of the $\theta_{j}$ are set to be 0 and 1, respectively, for scale identification. Second, the constraint $g_{i}<1-s_{i}$ is set to ensure that, even if guessing or slipping occurs, students who lack one or more required attributes have a lower probability of success than those who master all the required attributes (Junker and Sijtsma 2001). It should be noted that once an item parameter is decomposed as a linear combination of the item features (e.g., $\log it(s_{i})=\varphi_{0}+\sum_{m=1}^{M}\varphi_{m}Z_{im}$), it is hard to control the range of this parameter (e.g., $s_{i}$) as it is jointly affected by multiple item features; thus, the constraint $g_{i}<1-s_{i}$ has to be achieved by imposing a constraint on the other item parameter that is not linked with item features (e.g., $g_{i}<1-\frac{\exp(\varphi_{0}\;+\;\sum_{m=1}^{M}\varphi_{m}Z_{im})}{1\;+\;\exp(\varphi_{0}\;+\;\sum_{m=1}^{M}\varphi_{m}Z_{im})}$). This implies the technical challenge in imposing the constraint of $g_{i}<1-s_{i}$ while linking the item features to the guessing and slipping parameters simultaneously.

For those interested in explaining both guessing and slipping parameters with item features, a viable strategy would be using a two-step procedure: (1) estimate the item guessing or slipping parameters from the HO-DINA model and (2) regress the guessing and slipping estimates on the item features. However, one possible trade-off of such two-step procedure is that the measurement error in the parameters from the HO-DINA model in Step 1 may carry over to Step 2 to impact the regression coefficient estimation.

## 4. Empirical Data Analysis

### 4.1. Data

The proposed IE-HO-DINA models were applied to the released 2011 TIMSS data with a focus on the sample of the United States fourth-grade in the math domain. To maximize the number of items in the current analyses, we used a total of 37 items from two released math booklets, Booklets 5 and 6. The two booklets had 14 items in common. A sample of 1802 participants who had complete responses to either of the booklets were used.

The items were designed to measure three content domains, including number, geometric shapes and measures, and data display (Foy et al. 2013) and these content domains were treated as attributes in the Q-matrix specification (See Table S1 in the Online Supplementary Materials). All the item scores were dichotomized.

### 4.2. Feature Engineering

Item features used in this study include one item type feature and twenty-three item linguistic features (See Tables S2 and S3 in Online Supplementary Materials), based on findings from previous studies investigating linguistic features of assessment items (e.g., Drum et al. 1981; Paap et al. 2015). Most linguistic features were extracted with text mining techniques using Python 2.7.10 (Python Software Foundation 2015). The linguistic feature engineering was divided into two processes, text preprocessing and feature extraction, which are explained below.

#### 4.2.1. Text Preprocessing

Text in the item stems was organized into a plain text document with mathematical symbols removed, which ensured that only text corpus to be mined. The corpus of each item was disassembled into individual words (i.e., unigrams) or sentences, which served as units of analysis in the feature extraction.

#### 4.2.2. Feature Extraction

The linguistic features used in this study were roughly divided into three categories according to their extraction strategies: the features based on raw tokens, the features based on the part-of-speech tagging, and the features based on word lists. First, the features based on raw tokens (e.g., the number of words and the number of sentences) refer to the summary statistics (e.g., count, length) of the tokens. Second, the features based on the part-of-speech tagging (e.g., the number of verbs, the number of adjectives) were created as follows. The word tokens were labeled with their parts of speech (e.g., noun, verb, adjective, adverb) corresponding to the context of each sentence with the Python nltk package (Bird and Loper 2004). The characteristics of these labeled tokens were summarized by each item (e.g., number of verbs, number of nouns). To create features for the last category, we imported three standard word lists into Python as references, including the Dale–Chall word list (Dale and Chall 1955), the Brown News word list and the function word list. The Dale–Chall word list uses a list of about 3000 words that groups of fourth-grade American students could reliably understand, considering any word not on that list to be difficult. This word list was developed in a readability test that provides a numeric gauge of the comprehension difficulty that readers come upon when reading a text (Dale and Chall 1955). The Brown News word list, containing a total of 100,554 words, is a part of the Brown Corpus. The Brown Corpus is a general text collection containing 500 samples of English text compiled at the Brown University. The Brown Corpus was imported with the nltk.corpus Python module (Bird and Loper 2004). The function word list contains 277 words that express grammatical relationships in sentences (e.g., “*almost*” and “*even*”) retrieved from an open-access online resource (“Function word lists”, 2013), which is an updated version of the function word list compiled by O’Shea et al. (2012). In this feature category, the item features were extracted according to the presence of tokens in a word list.

Given that the IE-HO-DINA models contain a linear regression component, multicollinearity can yield unstable regression coefficient estimates (Farrar and Glauber 1967). Thus, the correlations among the item features were examined before conducting the analyses. It was found that some linguistic features (e.g., word token and the number of sentences) were highly correlated (ρ>0.9). To reduce the effect of multicollinearity, only eight features that were weakly inter-correlated (ρ<0.3) were retained in the analyses (see Table S2).

The descriptive statistics of the eight features based on the 37 analyzed items are summarized in Table S4 in the Online Supplementary Material. The variance inflation factors of all eight features were lower than 5, which suggested no evidence of multicollinearity (Craney and Surles 2002).

### 4.3. Model Estimation

The four proposed models were fit to the data. In addition, the HO-DINA model was fitted to the data as a baseline for comparison. When the data-fitting model was the HO-DINA model, the guessing and slipping estimates were then regressed on the item features (i.e., the two-step procedure) and the resulting item feature estimates were compared with those from the proposed IE-HO-DINA models.

The parameters of the HO-DINA and the proposed IE-HO-DINA models were estimated with the Bayesian MCMC method. The parameter estimation was conducted using JAGS 4.2.0 (Plummer 2015), which is called from R 3.4.3 (R Development Core Team 2013) with the R2jags package v0.5-7 (Su and Yajima 2015).

Below, we elaborate the prior distributions, joint posterior distribution, and full conditional distributions of the model parameters. The IE-HO-DINA model parameters had similar prior settings to those in the HO-DINA model except the guessing and slipping parameters. The prior distributions of the attribute mastery probability, guessing, slipping and higher-order structural parameters in the HO-DINA model were specified as:

$$a_{jk}|\theta_{j},\beta_{k},\xi_{k}∼\mathrm{Bernoulli}\left(\frac{\exp\left(\xi_{k}\theta_{j}+\beta_{k}\right)}{1+\exp\left(\xi_{k}\theta_{j}+\beta_{k}\right)}\right),$$

$$\theta_{i}∼\mathrm{Normal}\left(0,1\right),$$

$$\beta_{k}∼\mathrm{Normal}\left(0,2\right),$$

$$\xi_{k}∼\mathrm{Normal}\left(0,2\right)I\left(0,\right),$$

$$g_{i}∼\mathrm{Beta}\left(1,1\right),$$

$$s_{i}∼\mathrm{Beta}\left(1,1\right)I\left(,1-g_{i}\right).$$

In the IE-HO-DINA-g model, the prior distributions of the item feature coefficients were set as

$$\gamma_{0}∼\mathrm{Normal}\left({0,10}^{6}\right),\;\gamma_{m}∼\mathrm{Normal}\left({0,10}^{6}\right).$$

In the IE-HO-DINA-g-R model, the prior distribution of the guessing parameter was set as

$$\mathrm{logit}\left(g_{i}\right)∼N\left(\gamma_{0}+\sum_{n=1}^{M}\gamma_{m}Z_{im},\sigma_{\varepsilon }^{2}\right),\;s_{i}∼\mathrm{Beta}\left(1,1\right)I\left(,1-g_{i}\right).$$

where $\sigma_{\varepsilon }^{2}∼\mathrm{InvGamma}\left(1,1\right)$. The priors of the IE-HO-DINA-s and IE-HO-DINA-s-R model parameters could be set in a similar manner to those in the IE-HO-DINA-g and IE-HO-DINA-g-R models.

The joint posterior distribution of the IE-HO-DINA-s-R model parameters is

$$P\left(s,g,\alpha ,\gamma ,\theta ,\xi ,\beta ,\sigma_{\varepsilon }^{2}|Y,Z\right)\propto L\left(s,g;\alpha \right)P\left(g|Z,\gamma ,\sigma_{\varepsilon }^{2}\right)P\left(\gamma \right)P\left(\sigma_{\varepsilon }^{2}\right)P\left(s\right)P\left(\alpha |\theta ,\xi ,\beta \right)P\left(\theta \right)P\left(\xi \right)P\left(\beta \right)$$

Note that the joint posterior and full conditional distributions for the IE-HO-DINA-g model are largely similar to those of the IE-HO-DINA-g-R model, with the key difference being the absence of the error variance term, $\sigma_{\varepsilon }^{2}$, in the IE-HO-DINA-g model.

The full conditional distributions of the IE-HO-DINA-g-R parameters given the data and the other parameters are

$$P\left(\gamma |Y,Z,s,g,\alpha ,\theta ,\xi ,\beta ,\sigma_{\varepsilon }^{2}\right)\propto P\left(g|Z,\gamma ,\sigma_{\varepsilon }^{2}\right)P\left(\gamma \right)$$

$$P\left(\sigma_{\varepsilon }^{2}|Y,Z,s,g,\alpha ,\gamma ,\theta ,\xi ,\beta ,\sigma_{\varepsilon }^{2}\right)\propto P\left(g|Z,\gamma ,\sigma_{\varepsilon }^{2}\right)P\left(\sigma_{\varepsilon }^{2}\right)$$

$$P\left(g|Y,Z,s,\alpha ,\gamma ,\theta ,\xi ,\beta ,\sigma_{\varepsilon }^{2}\right)\propto L\left(s,g;\alpha \right)P\left(g|Z,\gamma ,\sigma_{\varepsilon }^{2}\right)$$

$$P\left(s|Y,Z,g,\alpha ,\gamma ,\theta ,\xi ,\beta ,\sigma_{\varepsilon }^{2}\right)\propto L\left(s,g;\alpha \right)P\left(s\right)$$

$$P\left(\alpha |Y,Z,s,g,\gamma ,\theta ,\xi ,\beta ,\sigma_{\varepsilon }^{2}\right)\propto L\left(s,g;\alpha \right)P\left(\alpha |\theta ,\xi ,\beta \right)$$

$$P\left(\xi |Y,Z,s,g,\alpha ,\gamma ,\theta ,\beta ,\sigma_{\varepsilon }^{2}\right)\propto P\left(\alpha |\theta ,\xi ,\beta \right)P\left(\xi \right)$$

$$P\left(\beta |Y,Z,s,g,\alpha ,\gamma ,\theta ,\xi ,\sigma_{\varepsilon }^{2}\right)\propto P\left(\alpha |\theta ,\xi ,\beta \right)P\left(\beta \right)$$

$$P\left(\theta |Y,Z,s,g,\alpha ,\gamma ,\xi ,\beta ,\sigma_{\varepsilon }^{2}\right)\propto P\left(\alpha |\theta ,\xi ,\beta \right)P\left(\theta \right)$$

Two chains with lengths of 20,000 were run and the first 10,000 iterations of each chain were discarded as burn-in. The potential scale reduction factor (PSRF; Brooks and Gelman 1998) and the trace plots were checked to assess convergence. The PSRF of the parameters were all lower than 1.1 and the trace plots have showed good mix of the two chains (example trace plots can be found in Figure S1 in Supplementary Online Material), which indicated that convergence has been achieved (Brooks and Gelman 1998).

## 5. Results

**Model fit.** The posterior predictive model check (Guttman 1967; Rubin 1981, 1984) was conducted to evaluate the data-model fit. The posterior predictive *p*-value (PPP) of the sum of squares of standardized residuals, which is a discrepancy measure between the data and the model, was calculated. Extremely small PPP value indicates a bad fit and this study regards PPP < 0.05 as a sign as bad model–data fit. Additionally, deviance information criterion (DIC; Spiegelhalter et al. 2002) was used to evaluate relative model fit. According to the PPP values shown in Table 1, all the five data-fitting models show an acceptable model–data fit. DIC results indicate that the IE-HO-DINA models (i.e., those without a residual term) are worse in model–data fit than the HO-DINA model or the IE-HO-DINA-R models, which is possibly due to the imperfect prediction of the item parameters from the item features. In contrast, the IE-HO-DINA-R models (i.e., those with a residual term) fit the data better than the HO-DINA model. The possible reason could be that, while the likelihood of the HO-DINA model and the IE-HO-DINA-R models were expected to be comparable, the IE-HO-DINA-R models contain fewer parameters than the HO-DINA model and, thus, could be less penalized for model complexity.

**The relationship between item features and item parameters.** $\gamma_{m}$ and $\varphi_{m}$ coefficients (Table 2 and Table 3) quantify the relationships between the item features and item parameters. In this study, the item features explained around 26% and 30% of the variance in the logit of the guessing and slipping parameters, respectively. The Wald test was performed to examine the null hypothesis that the parameters, $\gamma_{m}$ or $\varphi_{m}$, equals to 0. Only the “proportion of words with 6 or more letters” feature is statistically significant based on all the models. Specifically, this feature is negatively related to the guessing parameter but positively related to the slipping parameter.

The IE-HO-DINA-g or IE-HO-DINA-s model yields more statistically significant coefficients compared to the IE-HO-DINA-g-R model, the IE-HO-DINA-s-R model, or the two-step procedure. This could result from the fact that the standard errors of coefficients from the IE-HO-DINA-g or IE-HO-DINA-s model are only around 10% of those from the IE-HO-DINA-g-R model, the IE-HO-DINA-s-R model, or the two-step procedure.

**Consistency of item parameter estimates and attribute profile classifications.** The estimated guessing or slipping parameters from the HO-DINA model are highly correlated (correlation coefficient close to 1) with the predicted guessing or slipping parameters from the IE-HO-DINA-R models, but only moderately correlated (correlation coefficient ranging from 0.4 to 0.7) with those from the IE-HO-DINA models (i.e., those without residual terms). Accordingly, the attribute profile classifications from the HO-DINA model are highly consistent (consistency rate > 0.95) with those from the IE-HO-DINA-R models but relatively inconsistent (consistency rate at around 0.6) with the IE-HO-DINA models (i.e., those without residual terms). The item parameter correlation and attribute classification consistency among the models are listed in Tables S5 and S6 in the Online Supplementary Materials.

### A Simulation Study

In the empirical data analysis above, one of the major potential sources of misspecification of the proposed models is the misspecification of the explanatory part, i.e., the number of item features could be over-specified or under-specified. Therefore, this simulation study aims to examine the validity of the empirical data analysis results by investigating the impact of the misspecification of the explanatory component and, particularly, addressing two specific research questions (RQs): (1) How are the recoveries of feature coefficients, item parameters, and attribute profiles affected by the over-specification of the item features? (2) How are the recoveries of feature coefficients, item parameters, and attribute profiles affected by the under-specification of the item features?

The research questions were addressed under a scenario mimicking the empirical study: Twenty-five response datasets with 37 items measuring three attributes were generated. The Q-matrix remained the same as the one in the empirical data analysis. The response data were generated with an HO-DINA model. The true guessing and slipping parameters were both linear combinations of four simulated features along with some residual terms, i.e., $\log it(g_{i})=\psi_{0}+\sum_{m=1}^{4}\psi_{m}Z_{im}+\varepsilon_{(g)i}$ and $\log it(s_{i})=\varphi_{0}+\sum_{m=1}^{4}\varphi_{m}Z_{im}+\varepsilon_{(s)i}$. The simulated features can be either continuous or dichotomous, and be either strongly ($\left|\psi_{m}\right|$ or $\left|\varphi_{m}\right|$ = 0.6) or weakly ($\left|\psi_{m}\right|$ or $\left|\varphi_{m}\right|$ = 0.3) associated with the item parameters. The data-generating item features have explained approximately 60% of the variance in the true item parameters. The feature labels, true data generation model, item feature coefficients are listed in Table 4. The resulting true guessing and slipping parameters range from 0 to 0.5 and, thus, the simulated items consist of both high-quality (1-*s*-*g* ≥ 0.65) and low-quality (1-*s*-*g* < 0.65) items.

Different sets of models were fit to the simulated datasets to address different research questions, as articulated in Table 5. The impact of the over-specification of the explanatory component (RQ1) was examined by comparing the parameter recoveries from the correctly specified model against five over-specified models (each of the four proposed models and the two-step procedure had an over-specified version). Since item features were linked to both the slipping and guessing parameters in the data-generating model, the item feature coefficients in the correctly specified model had to be estimated with a two-step procedure since the IE-HO-DINA models cannot have item features linked to both guessing and slipping parameters simultaneously. In the over-specified models, four superfluous features in addition to the four data-generating features (i.e., a total of eight features) were linked to either guessing or slipping parameters. Details of the four superfluous item features are also listed in Table 4.

The impact of the under-specification of the explanatory component (RQ2) was examined by comparing the parameter recoveries from the correctly specified model against four under-specified models. As the residual terms in the IE-HO-DINA-R models have absorbed the unexplained variance in the item parameters, the under-specification of the item feature was expected to have little impact on the recoveries on the IE-HO-DINA-R models. Therefore, the impact of under-specification was only examined for the IE-HO-DINA models that have no residual term. Two types of under-specified models were considered: the IE-HO-DINA-2-strong models only retained the “strong” features (i.e., features with $\left|\psi_{m}\right|$ or $\left|\varphi_{m}\right|$ = v0.6) and ignored “weak” features (i.e., features with $\left|\psi_{m}\right|$ or $\left|\varphi_{m}\right|$ = 0.3) in the data-generating model; the IE-HO-DINA-2-weak models only retained the “weak” features and ignored “strong” features in the data-generating model. Features in the IE-HO-DINA-2-strong and IE-HO-DINA-2-weak models have explained approximately 40% and 15% of the variance in the item parameters, respectively.

The recovery of the continuous model parameters (i.e., item feature coefficients and item parameters) was evaluated in terms of bias and root mean squared error (RMSE). Specifically, $Bias(y)=\frac{1}{R}\sum_{r=1}^{R}\hat{y}-y_{true}$ and $RMSE(y)=\sqrt{\frac{1}{R}\sum_{r=1}^{R}{\left(\hat{y}-y_{true}\right)}^{2}}$, where *y* is the parameters to be evaluated and *R* is the number of replications. The recovery of the binary attribute parameters was evaluated in terms of the profile correct classification rate (PCCR) and the attribute correct classification rate (ACCR).

## 6. Results

**Figure 1:**
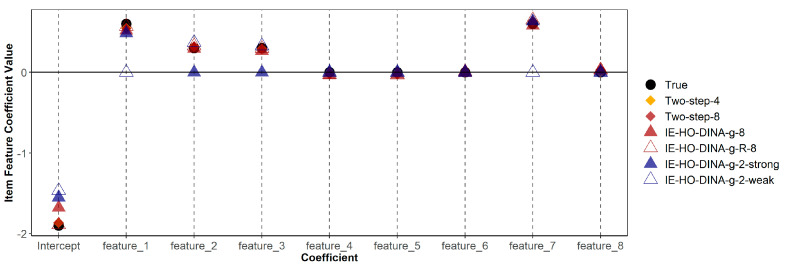
True and estimated item feature coefficients with guessing parameter as outcome. Two-step-4 = two-step procedure with the 4 data-generating features; Two-step-8 = two-step procedure with all 8 simulated features; IE-HO-DINA/IE-HO-DINA-R-8 = IE-HO-DINA/IE-HO-DINA-R model with all 8 simulated features; IE-HO-DINA-2-strong = IE-HO-DINA with only the 2 strong data-generating features; IE-HO-DINA-2-weak = IE-HO-DINA with only the 2 weak data-generating features.

**Figure 2:**
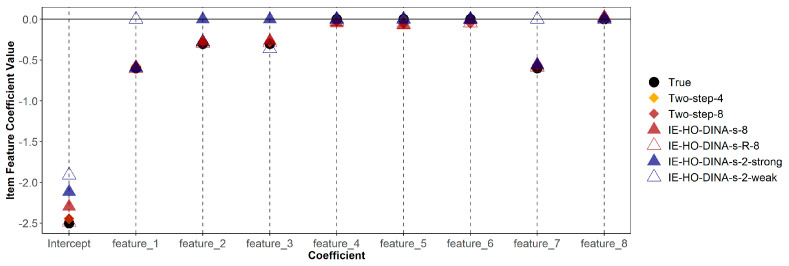
True and estimated item feature coefficients with slipping parameter as outcome.

Table 6 demonstrates that misspecified models (both over-specified and under-specified) do not consistently show poorer recovery (in terms of bias or RMSE) of item feature coefficients compared to the correctly specified model. Additionally, Figure 1 and Figure 2 indicate that estimates of guessing/slipping feature coefficients are similar across the correctly specified and misspecified models. As shown in Figure 1, in the over-specified models (IE-HO-DINA-g-8 and IE-HO-DINA-g-R-8), coefficients of superfluous features (features 4, 5, 6, and 8) are observed to be near zero. Conversely, in the under-specified models (IE-HO-DINA-g-2-strong and IE-HO-DINA-g-2-weak), despite the omission of certain data-generating features (i.e., omitting features 2 and 3 for IE-HO-DINA-g-2-strong and omitting features 1 and 7 for IE-HO-DINA-g-2-weak), the remaining feature coefficients closely approximate the true values. Nevertheless, the intercept estimates from the under-specified models show greater deviation from the true value than the other models. A similar pattern is observed for the slipping feature coefficients in Figure 2.

The item parameter recoveries are summarized in Table 7. Among the models without a residual term, the over-specified model (IE-HO-DINA-8) outperformed the under-specified models (IE-HO-DINA-2-strong and IE-HO-DINA-2-weak) in item parameter recovery. Although the magnitude of bias is comparable across these models, the RMSE is higher in the under-specified models. This suggests that an increase in unexplained variance in the item parameters may lead to greater random error in the item parameter estimates. However, compared to the model with a residual term (IE-HO-DINA-8-R) and the HO-DINA model, the model without a residual term (IE-HO-DINA-8) exhibits a larger RMSE. This increase in RMSE might be attributed to a significant portion of variance in item parameters remaining unexplained, even with the inclusion of 8 features. Incorporating a residual term could help absorb the unexplained variance in the item parameters, thereby potentially reducing the random error in item parameter estimates.

**Figure 3:**
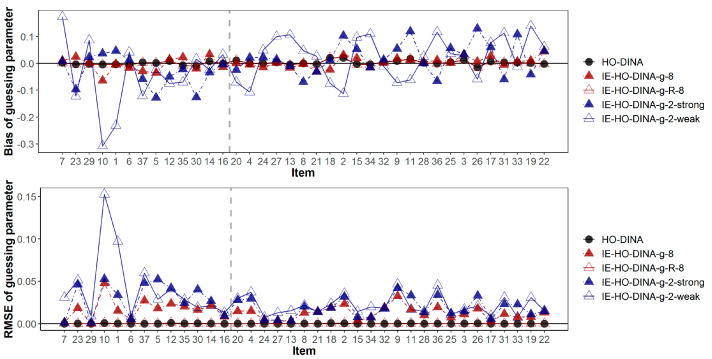
Bias and root mean squared error of guessing parameter estimates. Items are ascendingly ordered by their item quality (i.e., the true value of 1-s-g). The vertical gray dashed line separates the low- and high-quality items. Items to the left of the gray dashed line are of low quality (1-s-g < 0.65), while items to the right of the gray dashed line are of high quality (1-s-g ≥ 0.65).

**Figure 4:**
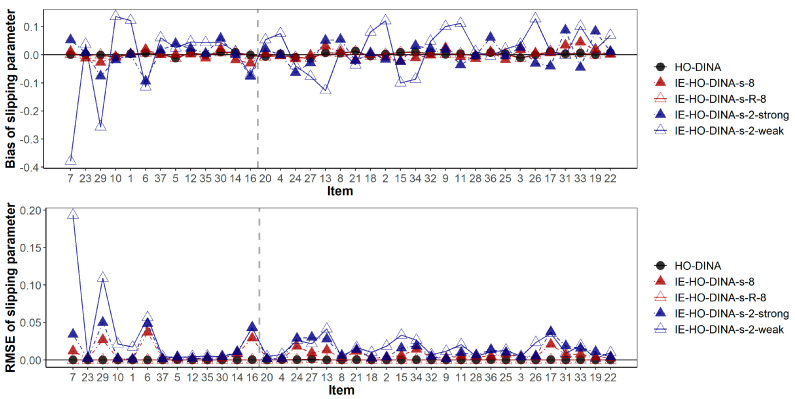
Bias and root mean squared error of slipping parameter estimates. Items are ascendingly ordered by their item quality (i.e., the true value of 1-s-g). The vertical gray dashed line separates the low- and high-quality items. Items to the left of the gray dashed line are of low quality (1-s-g < 0.65), while items to the right of the gray dashed line are of high quality (1-s-g ≥ 0.65).

In addition, Figure 3 and Figure 4 have demonstrated the item-wise guessing/slipping parameter recoveries where the items are ascendingly ordered by their true item quality (quantified by 1-*s*-*g*) on the x-axis. On average, the guessing/slipping parameters of the low-quality items (items with true 1-*s*-*g* < 0.65) have higher absolute bias and RMSE than the high-quality items (items with true 1-*s*-*g* ≥ 0.65).

As for the attribute classification accuracy shown in Table 8, the HO-DINA model, the over-specified models, and the IE-HO-DINA-2-strong models have achieved high attribute classification accuracy (PCCR and ACCRs > 0.9). Only the IE-HO-DINA-2-weak models have displayed lower attribute classification accuracy (PCCR and ACCRs ≤ 0.9) than the other models. These results have suggested that the imperfect prediction of the item parameters from the item features may not significantly diminish the attribute classification accuracy until a sufficiently large proportion of variance in the item parameters is left unexplained.

## 7. Summary and Discussion

Understanding the explanatory relationship between the item parameters and item features could help item developers discover the cause of the low-quality items (e.g., items with high guessing or slipping probabilities) and devise plans to revise them. The rapid advance of NLP and machine learning techniques has rendered it possible to extract more complex item features automatically and efficiently, thereby increasing the feasibility and usefulness of the proposed item explanatory CDMs.

The utility of the proposed models was demonstrated with the TIMSS released items and response data: around 20 item linguistic features were extracted with the NLP techniques; the proposed models were used to examine the relationships between the guessing/slipping parameters of the HO-DINA model and eight of the item features.

However, while the proposed models in this study aim to shed light on the relationship between item parameters and features, their inferences should not dictate item development practices deterministically. Instead, inferences from the models are intended to guide item developers by highlighting potential issues and areas for improvement. For instance, statistically significant features identified by the model can inform prioritization in item revision plans. In the case of reducing an item’s slipping probability, if the model indicates that “the proportion of words with 6 or more letters” significantly affects slipping, developers might first focus on modifying complex word proportions in the item stem. However, this focus on statistically significant features should not preclude consideration of other aspects such as item length. Additionally, from a score validity perspective, the proposed models can aid in uncovering sources of construct-irrelevant variance, such as the potential impact of complex wording on slipping effects. Ultimately, the model’s insights should complement, not replace, expert judgment in item development and revision processes.

The validity of the empirical data analysis results was further corroborated by a follow-up simulation study that mimicked the setting of the empirical data. The results from the simulation study have supported that, even with some slight misspecifications in the explanatory part of the proposed model, satisfactory recoveries in the item feature coefficients could be achieved. However, when a significant portion of variance in item parameters remains unexplained by the item features in item explanatory CDMs without a residual term, the recovery of the item parameters and attribute profiles may be compromised. Therefore, we recommend including a residual term in the item explanatory CDMs to enhance the accuracy of the model parameter estimates.

This study could be further extended in several directions. First, while this study has circumvented the multicollinearity issue by only including the weakly correlated features in the models, future studies could consider some modeling techniques which are robust to multicollinearity, such as the mean centering the variables (Iacobucci et al. 2016) and ridge regression (Hoerl and Kennard 1970), so that some potentially important features will not have to be eliminated. Moreover, key and distractor feature other than the item stem features could be included in the model as well.

Second, although this study has used the item features to explain only the guessing and slipping parameters of the HO-DINA model, it is straightforward to extend the proposed models to more generalized CDMs including the G-DINA model (de la Torre 2011), the LCDM (Henson et al. 2009) and the GDM (von Davier 2005). In particular, once the appropriate item features are extracted, they can be incorporated in the CDMs to explain the item parameter(s) of interest through a regression-like component. Further, the item features could be useful to explain the differential item functioning (DIF). For instance, if an item is detected to function differently across different subpopulations, the cross-group item parameter difference could be linked to the item features to investigate whether the DIF is associated with any item features, thereby facilitating the understanding of the cause of DIF.

Third, enhancing the computational efficiency of the model estimation is crucial for broader research and application of the proposed models. Currently, running the IE-HO-DINA and IE-HO-DINA-R models, with two MCMC chains of 10,000 iterations each, requires approximately 6 h and 30 h, respectively.^1^ This computational demand could limit more extensive explorations. Given that the ECDMs developed by Park et al. (2018), which include covariates on the person side, can be estimated using the expectation-maximization (EM) algorithm, future research could investigate the feasibility of adapting the EM algorithm for estimating parameters in the proposed models which have covariates on the item side.

Fourth, future research could consider varying sample sizes, test lengths, and Q-matrix specifications to enhance the generalizability of the simulation study. Additionally, investigating the impact of multicollinearity in item features on the inferences from item explanatory CDMs would be valuable. The scope of the current simulation study was limited by the substantial time required to run the models, constraining the feasibility to conduct broader simulations. Future studies, when feasible, could aim to determine the optimal number of items and persons necessary for accurate model parameter estimation.

Last but not least, the proposed models have the potential to be applied to address “cold start” problem in the future. Specifically, the newly developed assessments could suffer from the lack of empirical response data for item calibration, which was described as the “cold start” issue by Settles et al. (2020). Settles et al. (2020) have predicted the item difficulty parameters in the Rasch model with the item linguistic features, thus helping mitigate the “cold start” issue in a high-stakes language assessment. Analogously, the proposed models along with extracted item features may be used to predict the item parameters of the CDMs. Unfortunately, the limitations of the example empirical dataset restricted our ability to fully demonstrate the proposed model’s effectiveness in addressing the cold-start problem. The dataset’s small size, comprising only 37 items, limits its capacity for robustly training a model to learn the relationship between item features and parameters. Additionally, the item features extracted accounted for only about 30% of the variance in item parameters, reducing their predictive power for new items. Future research with larger item banks and more sophisticated NLP features, such as Bidirectional Encoder Representations from Transformers (BERT; Devlin et al. 2019) features, could be more useful to evaluate the proposed models’ effectiveness in tackling the cold-start problem. For instance, training the explanatory models with a subset of items from a larger bank and then predicting parameters for the remaining items could be a viable approach. However, it is important to note that there is a potential trade-off between a model’s explanative power and its predictive accuracy. Advanced NLP features such as BERT embeddings may enhance prediction capabilities at the cost of reduced explainability, as these features are often complex and not easily interpretable. Therefore, we advise researchers to carefully balance the need for explanatory insight against predictive precision when selecting features for their models.

## Figures and Tables

**Table 1:** Data fitting models and model fit results in empirical data analysis.

Model	The Item Parameter Linked to Item Features	Contain a Residual Term or Not	# of Parameters	PPP	DIC
HO-DINA	-	-	80	0.455	44,515.42
IE-HO-DINA-g	Guessing	No	52	0.478	46,540.49
IE-HO-DINA-g-R	Guessing	Yes	53	0.454	44,499.50
IE-HO-DINA-s	Slipping	No	52	0.215	45,717.00
IE-HO-DINA-s-R	Slipping	Yes	53	0.441	44,394.05

*Note*. PPP = posterior predictive *p*-value; DIC = deviance information criterion.

**Table 2:** Regression coefficient and standard error estimates of guessing features.

Coefficient	Data Fitting Model					
	HO-DINA with Two-Step Procedure		IE-HO-DINA-g		IE-HO-DINA-g-R	
	Estimate	SE	Estimate	SE	Estimate	SE
Word token	−0.01	0.03	−0.01	<0.01	−0.01	0.02
Number of adjectives	−0.11	0.18	0.01	0.02	−0.12	0.17
Number of adverbs	−0.25	0.43	−0.18 *	0.06	−0.25	0.40
Story or not	0.09	0.45	0.18 *	0.05	0.09	0.42
Item type	0.49	0.45	0.72 *	0.06	0.48	0.38
Proportion of tokens with six or more letters	−4.76 *	2.34	−2.44 *	0.30	−4.64 *	2.12
Number of non-Dale–Chall words	0.01	0.10	0.03	0.02	0.01	0.08
Brown News popularity	<0.01	0.01	<0.01	<0.01	<0.01	0.01

*Note*. * *p* < .05.

**Table 3:** Regression coefficient and standard error estimates of slipping features.

Coefficient	Data Fitting Model					
	HO-DINA with Two-Step Procedure		IE-HO-DINA-s		IE-HO-DINA-s-R	
	Estimate	SE	Estimate	SE	Estimate	SE
Word token	<0.01	0.03	<0.01	<0.01	<0.01	0.03
Number of adjectives	0.17	0.18	0.18 *	0.03	0.17	0.15
Number of adverbs	0.29	0.42	0.34 *	0.05	0.38	0.40
Story or not	−0.46	0.43	−0.79 *	0.08	−0.55	0.39
Item type	−0.46	0.45	−0.44 *	0.07	−0.50	0.37
Proportion of tokens with 6 or more letters	4.73 *	2.28	7.14 *	0.33	4.31 *	1.87
Number of non-Dale–Chall words	<0.01	0.10	0.09 *	0.02	0.02	0.09
Brown News popularity	<0.01	<0.01	<0.01	<0.01	<0.01	<0.01

*Note*. * *p* < .05.

**Table 4:** Specification of the simulated features.

Feature Label	Properties	True Data-Generating Model	$\psi_{m}$ ^a^	$\varphi_{m}$ ^a^
Feature 1	Continuous	Normal (0, 1)	0.6	−0.6
Feature 2	Continuous	Normal (0, 1)	0.3	−0.3
Feature 3	Continuous	Normal (0, 1)	0.3	−0.3
Feature 4	Continuous	Normal (0, 1)	0	0
Feature 5	Continuous	Normal (0, 1)	0	0
Feature 6	Continuous	Normal (0, 1)	0	0
Feature 7	Dichotomous	Bernoulli (*p* = 0.5)	0.6	−0.6
Feature 8	Dichotomous	Bernoulli (*p* = 0.5)	0	0

^a^ $\psi_{m}$ means coefficient regressing on the guessing parameter; $\varphi_{m}$ means coefficient regressing on the slipping parameter.

**Table 5:** Research questions and corresponding data fitting models.

Research Question	Correctly Specified Model	Misspecified Model
RQ1 (Over-specified)	Two-step-4 (or HO-DINA)	Two-step-8
		IE-HO-DINA-g-8
		IE-HO-DINA-s-8
		IE-HO-DINA-g-R-8
		IE-HO-DINA-s-R-8
RQ2 (Under-specified)	Two-step-4 (or HO-DINA)	IE-HO-DINA-2-g-strong
		IE-HO-DINA-2-s-strong
		IE-HO-DINA-2-g-weak
		IE-HO-DINA-2-s-weak

**Table 6:** Bias and RMSE of the item feature coefficient estimates.

Explanatory Component Specification Type	Model ^a^	Guessing Feature Coefficients ^b^		Slipping Feature Coefficients ^b^	
		Bias	RMSE	Bias	RMSE
Correctly specified	Two-step-4	-	0.03	0.03	0.03
Over-specified	Two-step-8	-	0.03	-	0.04
	IE-HO-DINA-8	−0.02	0.04	-	0.05
	IE-HO-DINA-R-8	-	0.04	−0.01	0.04
Under-specified	IE-HO-DINA-2-strong	−0.04	0.04	0.02	0.04
	IE-HO-DINA-2-weak	0.05	0.03	−0.02	0.04

*Note*. ^a^ Two-step-4 = two-step procedure with the 4 data-generating features; Two-step-8 = two-step procedure with all 8 simulated features; IE-HO-DINA/IE-HO-DINA-R-8 = IE-HO-DINA/IE-HO-DINA-R model with all 8 simulated features; IE-HO-DINA-2-strong = IE-HO-DINA with only the 2 strong data-generating features; IE-HO-DINA-2-weak = IE-HO-DINA with only the 2 weak data-generating features. ^b^ The recovery of the guessing feature coefficients is only applicable to the IE-HO-DINA-g/IE-HO-DINA-g-R models; the recovery of the slipping feature coefficients is only applicable to the IE-HO-DINA-s/IE-HO-DINA-s-R models. Bias values that approaches 0 (i.e., −0.01 < Bias < 0.01) are represented with “-”.

**Table 7:** Bias and RMSE of the predicted guessing/slipping parameters.

Explanatory Component Specification Type	Model	Guessing ^b^		Slipping ^b^	
		Bias	RMSE	Bias	RMSE
-	HO-DINA ^a^	0.002	<0.001	0.002	<0.001
Over-specified	IE-HO-DINA-8	0.001	0.014	0.001	0.008
	IE-HO-DINA-R-8	0.002	<0.001	<0.001	<0.001
Under-specified	IE-HO-DINA-2-strong	0.004	0.022	0.003	0.013
	IE-HO-DINA-2-weak	-0.002	0.029	0.006	0.023

*Note*. ^a^ The guessing/slipping parameters from the HO-DINA model were estimated instead of predicted, and the recovery these parameter estimates were used as the baseline. ^b^ The recovery of the predicted guessing probabilities is only applicable to the IE-HO-DINA-g/IE-HO-DINA-g-R models; the recovery of the predicted slipping probabilities is only applicable to the IE-HO-DINA-s/IE-HO-DINA-s-R models.

**Table 8:** Attribute and profile classification accuracy.

Explanatory Component Specification Type	Model	PCCR	ACCR		
			A1	A2	A3
-	HO-DINA	0.932	0.933	0.999	1.000
Over-specified	IE-HO-DINA-g-8	0.915	0.916	0.998	1.000
	IE-HO-DINA-s-8	0.922	0.923	0.998	1.000
	IE-HO-DINA-g-R-8	0.931	0.932	0.999	1.000
	IE-HO-DINA-s-R-8	0.931	0.932	0.999	1.000
Under-specified	IE-HO-DINA-2-g-strong	0.913	0.915	0.999	1.000
	IE-HO-DINA-2-s-strong	0.914	0.916	0.998	1.000
	IE-HO-DINA-2-g-weak	0.870	0.871	0.998	1.000
	IE-HO-DINA-2-s-weak	0.908	0.910	0.998	1.000

## Data Availability

Toy datasets and example model scripts can be found in https://github.com/mancyliao/ie_cdm.

## Note

1 The computing time is based on the analyses run on a desktop with Intel Core i7 CPU and 3.2 GHz processor. Multiple MCMC chains were run in parallel with multiple cores. The sample size and number of items setup is similar to that in the empirical data analysis section.

## Supplementary Materials

The following supporting information can be downloaded at: https://www.mdpi.com/article/10.3390/jintelligence12030032/s1, Figure S1: Example trace plots (post-burn-in iterations) of the item feature coefficients from the IE-HO-DINA-g and IE-HO-DINA-g-R models. (a) and (b) are from the IE-HO-DINA-g model; (c) and (d) are from the IE-HO-DINA-g-R model. Table S1: Q-matrix Used in the Analyses; Table S2: Descriptions of the Eight Item Features Used in the Analyses; Table S3: Item Features that were Created but not Included in the Analyses; Table S4: Descriptive Statistics of Item Features; Table S5: Correlation among the Predicted or Estimated Guessing/Slipping Parameters from the Data Fitting Models; Table S6: Attribute Profile Classification Consistency Among the Data Fitting Models.
