# Supplementary material for: Explanatory Cognitive Diagnosis Models Incorporating Item Features
Source: J Intell. 2024 Mar 11;12(3):32. doi: 10.3390/jintelligence12030032 (PMC10971092; doi:10.3390/jintelligence12030032)
Supplement: Supplementary file 1 [file jintelligence-12-00032-s001.zip › jintelligence-2571620-supplementary.pdf]

Explanatory Cognitive Diagnosis Models Incorporating Item Features

Online Supplementary Documents

Table S1

Q-matrix Used in the Analyses

Item	Number	Geometric shapes and measures	Data display
1	0	0	1
2	0	0	1
3	0	0	1
4	1	0	1
5	1	1	1
6	1	0	1
7	0	1	1
8	0	0	1
9	0	1	0
10	0	1	1
11	1	0	0
12	0	0	1
13	0	0	1
14	0	0	1
15	0	0	1
16	0	0	1
17	1	0	1
18	1	0	1
19	0	1	0
20	0	1	0
21	0	1	0
22	0	1	1
23	0	1	0
24	1	0	1
25	1	0	0
26	0	0	1
27	0	0	1
28	0	0	1
29	0	0	1
30	0	0	1
31	1	0	1
32	1	0	1
33	0	1	0
34	0	0	1
35	0	1	0
36	0	1	1
37	0	1	0

Table S2

Descriptions of the Eight Item Features Used in the Analyses

Feature label	Description
Word token[a]	The number of words excluding punctuation in the stem.
Number of adjectives	The number of adjectives in the stem.
Number of adverbs	The number of adverbs in the stem.
Story or not	Binary indicator of whether the item has a story context (1=Yes; 0=No).
Item type	Binary indicator of whether the item is a multiple choice item or a constructed response item (1=Multiple choice; 0=Constructed response).
Proportion of words with 6 or more letters[a]	The proportion of words with 6 or more letters in the stem.
Number of non Dale-Chall words[a]	The number of words that are not in the Dale-Chall word list. Dale-Chall word list contains around 3000 words that are most familiar to the fifth-grade children. When 80% of the fourth-graders who participated in the readability study indicated they knew a word, the word was added to the Dale-Chall word list using the Dale-Chall formula (Dale & Chall, 1948).
Brown News popularity[a]	The popularity of verbs, nouns, adjectives, adverbs, and names in the Brown News Corpus. Brown News Corpus is a reference database in natural language processing that contains a total of 100,554 words.

[a]Based on Drum et al. (1981); Lepik (1990); and Paap et al. (2015).

Table S3

Item Features that were Created but not Included in the Analyses

Feature label	Description
Number of letters (alphabet only)	The number of alphabet letters in the stem.
Number of letters (numbers only)	The number of numbers in the stem.
Number of letters (including alphabet and number)	The number of alphabet letters and numbers in the stem.
Number of letters (all included)	The number of letters including alphabet, number, space, punctuation, etc., in the stem.
Number of words (alphabet only)	The number of words in the stem, excluding numbers and units.
Number of unique words (Word type)	The number of words excluding word repetition and punctuation (i.e., Vocabulary size) in the stem.
Word diversity	The ratio of "word type" to "word token".
Number of verbs	The number of verbs in the stem.
Number of nouns	The number of nouns in the stem.
Number of content words	The number of content words in the stem. Content words include nouns, verbs (except *to be)*, adjectives and adverbs (except quantifiers or intensifiers).
Number of unique content words	The number of unique content words in the stem.
Proportion of content words	The proportion of content words in the stem (i.e., ratio of "number of content words" to "word token").
Number of function words	The number of function words in the stem. Function words serve to express grammatical relationships in sentence. Examples of function words are *almost*, *about* and *even*.
Number of words with 6 or more letters	The number of words with 6 or more letters in the stem.
Mean word length	The ratio of "Number of letters (alphabet only)" to "Number of words (alphabet only)".
Number of sentences	The number of sentences in the stem.

Table S4

Descriptive Statistics of Item Features

Feature name	Mean	SD	Min	Max
Word token	21.92	11.73	7	67
Number of adjectives	1.46	1.32	0	6
Number of adverbs	0.43	0.60	0	2
Story or not	0.54	-	0	1
Item type	0.57	-	0	1
Proportion of tokens with 6 or more letters	0.24	0.10	0.06	0.47

Number of non Dale-Chall words	4.54	3.24	0	13
Brown News popularity	31.79	24.00	6.50	140.50

Table S5

Correlation among the Predicted or Estimated Guessing / Slipping Parameters from the Data Fitting Models

Model	HO-DINA	IE-HO-DINA (without a residual term)
IE-HO-DINA (without a residual term)	0.42 / 0.70	
IE-HO-DINA-R	1.00 / 1.00	0.43 / 0.70

Note. The guessing and slipping parameters from the HO-DINA model are estimated values; The guessing and slipping parameters from the IE-HO-DINA or IE-HO-DINA-R models are predicted values based on the item features and feature coefficients. Values in the each cell indicate the correlations for the guessing / slipping parameters.

Table S6

Attribute Profile Classification Consistency Among the Data Fitting Models

Model	HO-DINA	IE-HO-DINA-g	IE-HO-DINA-g-R	IE-HO-DINA-s
IE-HO-DINA-g	0.65			
IE-HO-DINA-g-R	0.98	0.64		
IE-HO-DINA-s	0.58	0.37	0.59	
IE-HO-DINA-s-R	0.97	0.64	0.98	0.59

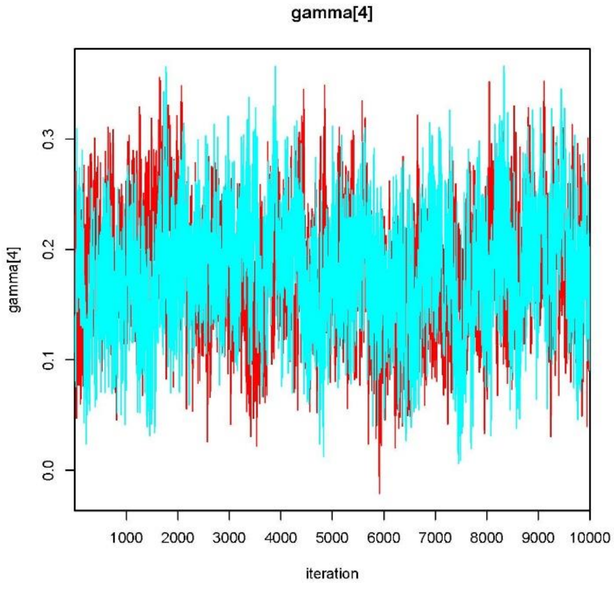

(a)

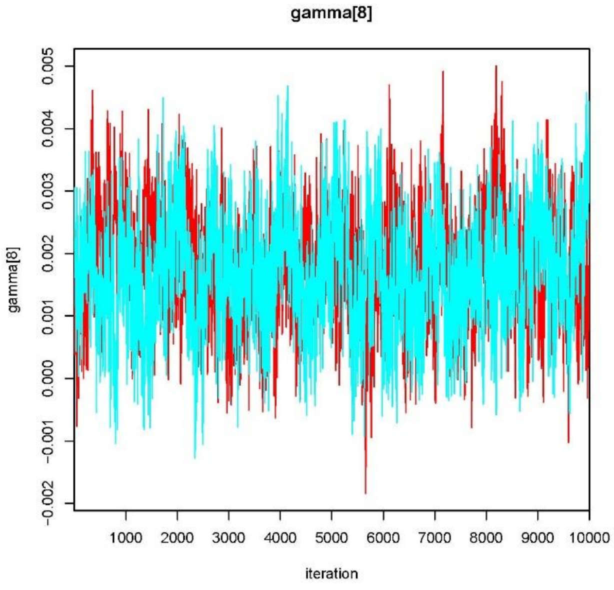

(b)

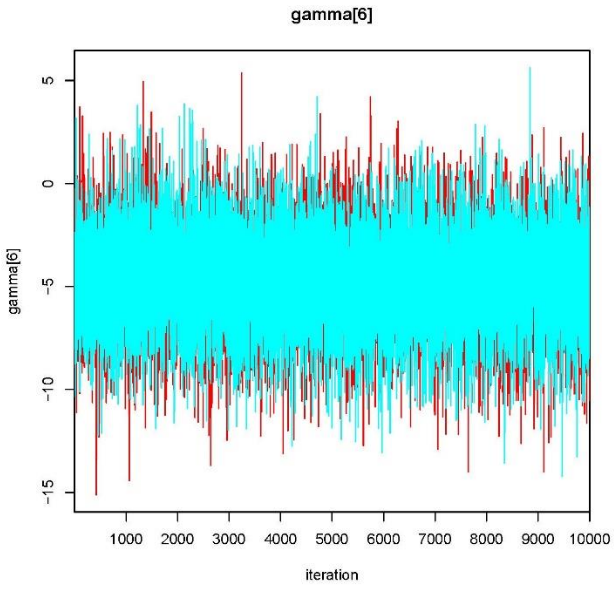

(c)

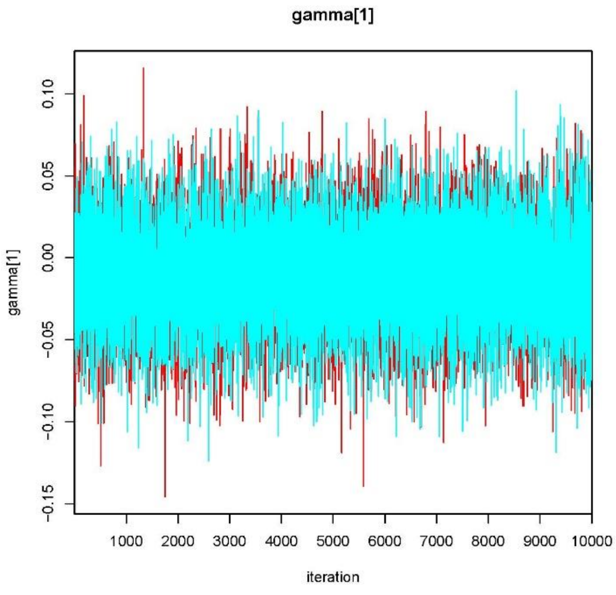

(d)

Figure S1. Example trace plots (post-burn-in iterations) of the item feature coefficients from the IE-HO-DINA-g and IE-HO-DINA-g-R models. (a) and (b) are from the IE-HO-DINA-g model; (c) and (d) are from the IE-HO-DINA-g-R model.
